# Increases in Brain ^1^H-MR Glutamine and Glutamate Signals Following Acute Exhaustive Endurance Exercise in the Rat

**DOI:** 10.3389/fphys.2017.00019

**Published:** 2017-01-31

**Authors:** Maciej Świątkiewicz, Michał Fiedorowicz, Jarosław Orzeł, Marlena Wełniak-Kamińska, Piotr Bogorodzki, Józef Langfort, Paweł Grieb

**Affiliations:** ^1^Department of Experimental Pharmacology and Laboratory of Nuclear Magnetic Resonance Imaging, Mossakowski Medical Research Centre, Polish Academy of SciencesWarsaw, Poland; ^2^Faculty of Electronics, Warsaw University of TechnologyWarsaw, Poland

**Keywords:** glutamate, glutamine, magnetic resonance spectroscopy, exercise, central fatigue, brain

## Abstract

**Objective:** Proton magnetic resonance spectroscopy (^1^H-MRS) in ultra-high magnetic field can be used for non-invasive quantitative assessment of brain glutamate (Glu) and glutamine (Gln) *in vivo*. Glu, the main excitatory neurotransmitter in the central nervous system, is efficiently recycled between synapses and presynaptic terminals through Glu-Gln cycle which involves glutamine synthase confined to astrocytes, and uses 60–80% of energy in the resting human and rat brain. During voluntary or involuntary exercise many brain areas are significantly activated, which certainly intensifies Glu-Gln cycle. However, studies on the effects of exercise on ^1^H-MRS Glu and/or Gln signals from the brain provided divergent results. The present study on rats was performed to determine changes in ^1^H-MRS signals from three brain regions engaged in motor activity consequential to forced acute exercise to exhaustion.

**Method:** After habituation to treadmill running, rats were subjected to acute treadmill exercise continued to exhaustion. Each animal participating in the study was subject to two identical imaging sessions performed under light isoflurane anesthesia, prior to, and following the exercise bout. In control experiments, two imaging sessions separated by the period of rest instead of exercise were performed. ^1^H-NMR spectra were recorded from the cerebellum, striatum, and hippocampus using a 7T small animal MR scanner.

**Results:** Following exhaustive exercise statistically significant increases in the Gln and Glx signals were found in all three locations, whereas increases in the Glu signal were found in the cerebellum and hippocampus. In control experiments, no changes in ^1^H-MRS signals were found.

**Conclusion:** Increase in glutamine signals from the brain areas engaged in motor activity may reflect a disequilibrium caused by increased turnover in the glutamate-glutamine cycle and a delay in the return of glutamine from astrocytes to neurons. Increased turnover of Glu-Gln cycle may be a result of functional activation caused by forced endurance exercise; the increased rate of ammonia detoxification may also contribute. Increases in glutamate in the cerebellum and hippocampus are suggestive of an anaplerotic increase in glutamate synthesis due to exercise-related stimulation of brain glucose uptake. The disequilibrium in the glutamate-glutamine cycle in brain areas activated during exercise may be a significant contributor to the central fatigue phenomenon.

## Introduction

Proton magnetic resonance spectroscopy (^1^H-MRS) *in vivo* enables non-invasive acquisition of certain information about brain biochemistry in animals and humans. A notable advantage of this technique over alternative approaches, such as *ex vivo* chemical assays of metabolites in tissue samples, is its non-invasive character which makes possible repeat measurements in the same subject, nullifying interindividual variability. Thanks to this feature MRS is a very suitable technique for monitoring metabolic changes due to disease and effects of treatment (van der Graaf, 2010). Its other important feature is “clinical translatability,” the possibility of investigating parallel experimental paradigms using animals and humans (Hermann et al., 2012).

In a frequently used ^1^H-MRS approach known as localized *in vivo* spectroscopy, magnetic field gradients are programmed to restrict magnetic resonance (MR) data acquisition to a discrete region (volume of interest, VOI) within an organ studied (Keevil, 2006). Clinical MR systems equipped with high field (1.5 T) magnets, which are currently widely available, enable reliable quantification of up to six resonance signals from the human brain: N-acetylaspartate (NAA), creatine and phosphocreatine (tCr), *myo-*inositol (Ins), lactate (Lac), a combined signal of several choline compounds (tCho), and a combined signal of glutamate and glutamine (Glx). Animal, and recently also human studies at ultra-high magnetic fields (≥7 T) demonstrated the feasibility of quantifying up to 18 brain metabolites, including glutamate (Glu) and glutamine (Gln) quantified separately *in vivo* (Öz et al., 2013; Rae, 2014).

Glutamate and glutamine are particularly important in the mammalian brain. Glutamate is not only the most abundant free amino acid, but also the main excitatory neurotransmitter. Being an essential constituent of most proteins, it must be readily available for protein synthesis. Another functions of glutamate in the brain are participating in nitrogen homeostasis as ammonia acceptor, and serving as a substrate for ATP production. Different functions of glutamate in the brain engage different glutamate pools, of which the glutamate pool serving neurotransmission appears to be the most labile and energy-consuming. On the basis of *in vivo* studies in which ^13^C-MRS was employed to measure the kinetics of changes in brain metabolite concentrations following injection or infusion of ^13^C-labeled substrates Rothman et al. (2003) concluded that both in humans and in rats 60–80% of total energy consumption in the non-stimulated cerebral cortex is used by processes linked to the glutamate neurotransmission.

As pointed out by Hertz et al. (1999), to make it possible to use glutamate as a neurotransmitter, several brain-specific “innovations” had to be worked out by evolution. One of them was separation of brain from blood with blood-brain barrier which tightly controls extracellular brain fluid composition irrespective of the level of glutamate in blood. The blood-brain barrier is poorly permeable to glutamate, thereby making the brain autonomous in the production of this amino acid. The second innovation was to surround glutamatergic synapses with astrocytes endowed with powerful glutamate carriers able to quickly remove glutamate from synaptic clefts, which is necessary to maximize the signal-to-noise ratio of neurotransmission. The third innovation pertained to compartmentation of glutamate amidating enzyme glutamine synthase to glia, enabling conversion of molecules of this neurotransmitter to molecules of glutamine devoid of neurotransmitter activity in order to return them to neurons—this is so-called glutamate-glutamine cycle. The other two concern the restriction to glial cells of the enzymes that are involved in the *de novo* synthesis from glucose of the carbon skeleton of glutamate, and the return of the carbon skeleton of excess glutamate to the metabolic pathway for glucose oxidation.

Brain activation during exercise is a well-known phenomenon which has been studied in awake, unrestrained animals with a variety of modalities. Methods such as electrophysiology, microdialysis (Wang et al., 2012), tracing enhancement of c-fos gene expression (an indirect marker of neuronal activity, Liste et al., 1997), and autoradiographic functional imaging (Holschneider and Maarek, 2008) have been used in these studies. Although the pattern of activation depends on experimental approaches (e.g., trained or untrained subjects, internal vs. external generation of movements, etc.), all these studies in general have shown that during exercise many brain structures are activated, including the cerebellum, basal ganglia, and motor cortex, see Holschneider et al. (2007) and the references quoted therein. The effects of exercise on various aspects of brain activity have also been studied in humans, see for example Boecker et al. (2010) and Hamacher et al. (2015). In particular, it has been observed that global brain energy turnover does not change during transition from rest to exercise or during low intensity exercise, whereas during more vigorous exercise an increased metabolic rate is detectable at the whole brain level (Nybo and Secher, 2004).

In spite of fundamental importance of glutamatergic neurotransmission in neurophysiology, the effects of exercise on the underlying biochemistry, namely on brain Glu-Gln cycle, have not been extensively studied to date. There were only a few reports on exercise-induced changes in ^1^H-MRS spectra *in vivo*, in which Glu, Gln, and/or Glx lines were recorded. In one human study (Maddock et al., 2011), ^1^H-MRS spectra from an 18.75 cm^3^ VOI (3 × 2.5 × 2.5 cm) centered on the bilateral primary visual cortices were recorded with a 1.5 T MR imaging and spectroscopy system, prior to, and immediately after graded exercise to ~85% of predicted maximum heart rate. This study presented no changes in NAA, Cho, and tCr, but significant rises in Lac and Glx (scaled to tCr signal). Twenty minutes after the end of exercise the ratio of Glx/tCr was increased by 17%. These increases, which appeared to be transient, were interpreted as a result of the activity-dependent increase in brain Glu content. However, in the other human study published recently Dennis et al. (2015) used a 7 T field MRS to acquire proton MR spectra from the visual cortex prior to and after short vigorous exercise in human volunteers, which made it possible to separate the signals of Glu and Gln. The result was different from that of Maddock et al. (2011), because there were no increase in either Glu or Gln normalized to tCr. Moreover, when the “absolute” quantification of the signals (i.e., normalization to the water signal) has been performed, both Glu and Gln were significantly lower after exercise. Also, in the animal experiment decreases of Glu and Glx resonance signals were found in 3.2 μL VOI (2.2 × 1.2 × 1.2 mm) placed in the right hippocampus of mice allowed to exercise voluntarily on a running wheel for 6–8 weeks, compared to control mice supplied with a blocked running wheel and not allowed to exercise (Biedermann et al., 2012). The divergent results of the aforementioned studies may reflect different metabolic responses to exercise of different strength and duration in different brain areas, difference between acute forced and chronic voluntary exercise, and/or difference between rodent and human brain.

The present study, performed on rats, aimed to determine change in proton MR signals from VOIs positioned in three brain regions engaged in motor activity caused by involuntary acute endurance exercise to exhaustion. The use of a small animal MR imaging system equipped with a 7 T magnet enabled the recording not only of the combined Glx resonance but also separate Glu and Gln signals. Whereas increase in the turnover rate of Glu-Gln cycle during intense exercise was expected, divergent results of the aforementioned ^1^H-MRS exercise experiments precluded formulation of working hypothesis concerning direction of the exercise-induced changes in Glu, Glx, and Gln resonance signals. However, we assumed that when the exercise will be conducted until exhaustion, the changes should be the most pronounced.

## Methods

### Animals

Eighteen male 6 weeks old Wistar rats (weighing between 240 and 260 g) from the inhouse animal breeding facility were used in this study. Rats were split into the study and control group. Between the first and the second MRS run 12 animals from the study group were exercising on a treadmill, whereas the additional six belonging to the control group were resting instead of exercising. The animals were housed 2–3 per cage, at 12/12 h light/dark cycles (lights on at 7:00 a.m.), 22–24°C ambient temperature, 45–65% relative humidity, and were provided with a standard diet and tap water *ad libitum*. Animal research followed internationally accepted guidance for the care and use of laboratory animals, including the National Institute of Public Health—National Institute of Hygiene (NIPH—NIH) guidelines, and was approved by the IV Warsaw Local Ethics Committee for Animal Experimentation in National Medicines Institute.

### Endurance exercise test

All experiments were performed between 10 a.m. and 1 p.m. Before the start of the MRS experiment rats were run-tested on a motorized BTP-10 rodent treadmill (Porfex, Bialystok, Poland; 18–20 m/min, 5, 10, and 15 min, with 15 min breaks) for 3 successive days to habituate them to treadmill running and to eliminate the individuals unwilling to run. In the habituating runs, to motivate the rats to continue running, negative reinforcement was used, i.e., animals were stimulated with low-intensity electrical shock (170 V, 0.5 mA alternate current). After a 3 day break, each of the 12 habituated rats performed a single bout of endurance exercise to exhaustion (60–90 min), on a flat treadmill running at 24 m/min (exercise group). Running parameters were chosen to keep the exercising rats around the anaerobic threshold, which for the untrained Wistar rat has previously been determined in our institution to occur at the treadmill speed of 25 m/min (Pilis et al., 1993). To avoid introduction of an additional stress, no negative reinforcement (e.g., electrical shocks) was used during running. The moment of exhaustion was taken when the exercising animal failed to keep up with the treadmill three times in a row during a period of <1 min.

### Magnetic resonance imaging sessions

**Figure 1:**
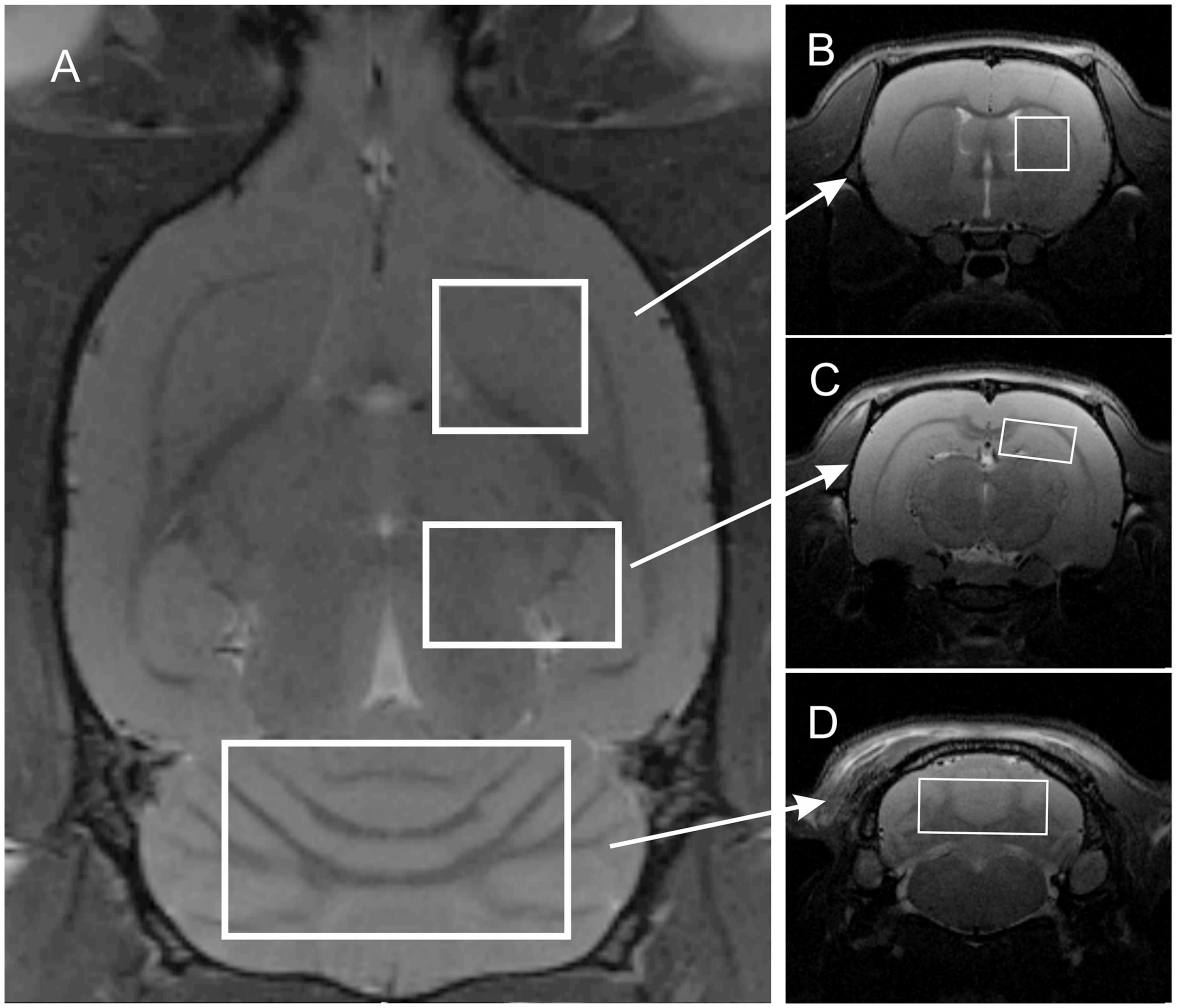
**Localization of three volumes of interest on T2-weighted images: (A)** axial slice; **(B–D)** coronal slices centered on the right striatum, right hippocampus, and cerebellum, respectively.

MR imaging and spectroscopy was performed with the 7 T Bruker BioSpec 70/30 Avance III system. Each of the 18 animals (exercise group and control group) participating in the study were subjected to two identical imaging sessions. The first was performed prior to and the second immediately following the exercise run or after 1 h 15 min for control group. Animals were anesthetized with 1.5–2% isoflurane in oxygen, and positioned head-first prone in the MR compatible animal bed. A transmit cylindrical radiofrequency coil (8.6 cm inner diameter) and a rat brain dedicated receive-only array surface coil (2 × 2 elements) were positioned over the animal's head. Respiration rate and body temperature were monitored throughout the experiment with a small animal monitoring system. Positioning tripilot scans were performed, followed by high resolution T_2_-weighted TurboRARE structural scan (T_R_/T_Eeff_ = 2500/33 ms, RARE factor = 8, spatial resolution = (117 × 117 × 700 μm), FOV = 30 × 30 mm, 22 slices, 400 μm gap, NEX = 2, Scan Time = 3 min) covering the whole brain. Using the high-resolution structural image, three VOIs were planned for subsequent MRS acquisitions with the following VOI dimensions: 3 × 3 × 3 mm for the striatum (Figures 1A,B), 7 × 3 × 4 mm for the cerebellum (Figures 1A,D), 4 × 2 × 2.5 mm for the right hippocampus (Figures 1A,C). Since all VOIs are cuboid in shape they encompass parts of other structures, but all were planned to maximize signal from the target brain structure and to minimize possible contamination by signals originating from neighboring structures.

Prior to spectra acquisition, for each of the predefined VOIs, an extensive shimming procedure was performed in order to maintain an unsuppressed water line width below 15 Hz, covering linear and second order global shims, followed by linear and second order local shimming with the FASTMAP protocol within the measured VOI. The shimming procedure lasted 15–20 min. Subsequently, the svMRS spectra were acquired with the short echo time PRESS sequence (TR/TE = 2000/20 ms, 512 averages, 2048 points, scan Time = 17 min) with VAPOR water suppression, the outer volume suppression, frequency drift correction (flip angle 5°) and eddy current correction.

### Spectra quantification and statistical analysis

Metabolite concentrations were estimated using the LCModel software (Provencher, 1993). The unsuppressed water signal measured from the same VOI as the metabolite spectrum is then employed as an internal reference for absolute quantification. The “standard” basis set for 7 T PRESS sequence with T_E_ 20 ms was used, containing the following metabolites: alanine (Ala), aspartate, (Asp), creatine (Cr), phosphocreatine (PCr), glycerophosphocholine (GPC), phosphocholine (PCh), γ-aminobutyric acid (GABA), glucose (Glc), glutamine (Gln), glutamate (Glu), glutathione (GSH), lactate (Lac), myo-Inositol (Ins), N-acetyl aspartate (NAA), N-acetyl aspartate glutamate (NAAG), scyllo-Inositol (Scyllo), and taurine (Tau). Relative standard error estimates of the resonance signals (Cramér-Rao lower bounds, CRLB) were used to establish the criterion for further analysis. Metabolites with mean CRLB ≤ 15% (excluding macromolecules) were identified and their respective concentrations were calculated from the spectra obtained from each VOI.

**Figure 2:**
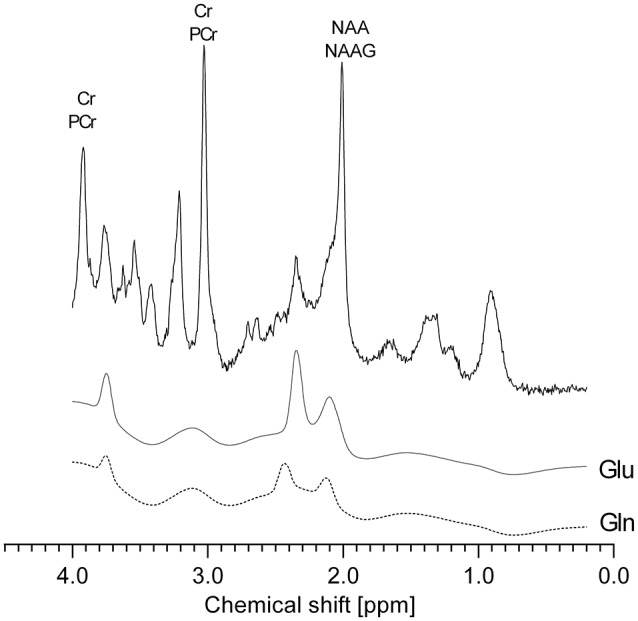
**A representative spectrum obtained from VOI localized in the rat cerebellum and an example of LCModel computation of metabolite concentrations**. The black solid line represents raw data, LCModel fits for glutamate and glutamine are shown below.

LCModel analysis provides absolute (molar) concentrations, however data variance may be inflated due to the difference caused by the partial volume effect (the relative contribution of tissue and CSF in a VOI). To decrease data variance, metabolite signals in the acquired spectrum were scaled to the signal of creatine and phosphocreatine (tCr) which in the brain appears to be stable within each tissue type, or to the water signal (Pouwels and Frahm, 1998). An example of the LCModel approximation of metabolite concentrations superimposed on the representative spectrum obtained from VOI localized in the rat cerebellum is showed in Figure 2.

The pre- and post-exercise data were tested with the Wilcoxon signed-rank test (a non-parametric test for repeated measurements) using GraphPad Prism version 6.04 for Windows (GraphPad Software, San Diego, CA, USA). Differences were considered significant when *p* < 0.05.

## Results

**Figure 3:**
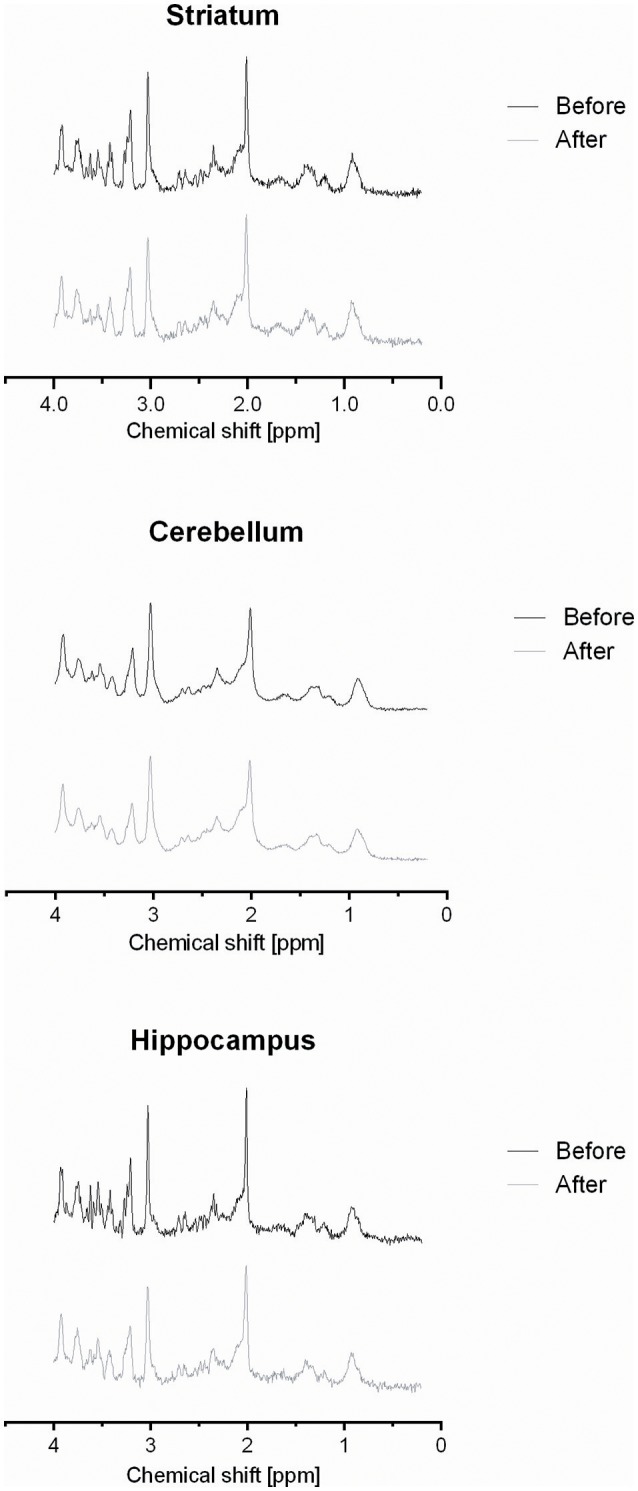
**Representative spectra obtained from VOIs localized in the rat striatum, cerebellum, and hippocampus before and after exercise**.

The VOIs centered at the right striatum, cerebellum and right hippocampus produced stable spectra with good signal to noise ratios (average SNRs 21.5, 40.3, and 21.9, respectively; Figure 3).

Analysis of the MR spectra revealed that after exercise Gln in the striatum was increased by 13% when scaled to tCr (*p* ≤ 0.001) and by 15% when scaled to water (*p* ≤ 0.001), and the Glx was increased by 7% (to tCr, *p* ≤ 0.005) and by 9% (to water, *p* ≤ 0.005), but there was no significant difference in Glu.

In the cerebellum Gln was increased by 32% when scaled to tCr (*p* ≤ 0.001) and by 34% when scaled to water (*p* ≤ 0.001), Glu was increased by 10% (to tCr *p* < 0.005), by 11% (to water, *p* ≤ 0.05) and Glx was increased by 17% (to tCr, *p* < 0.0005), by 18% (to water, *p* ≤ 0.001).

**Figure 4:**
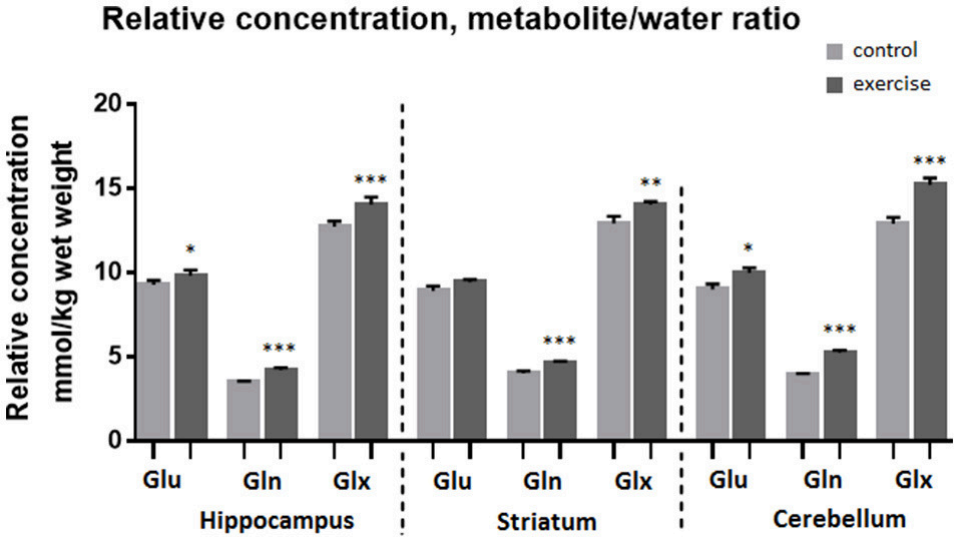
**Average relative concentration of Glu, Gln, and Glx in voxels centered on the hippocampus, striatum, and cerebellum before and after exercise**. Individual values of metabolite concentrations were scaled to water. Mean ± SEM. ^*^P < 0.05, ^**^P < 0.01, ^***^P < 0.001.

**Figure 5:**
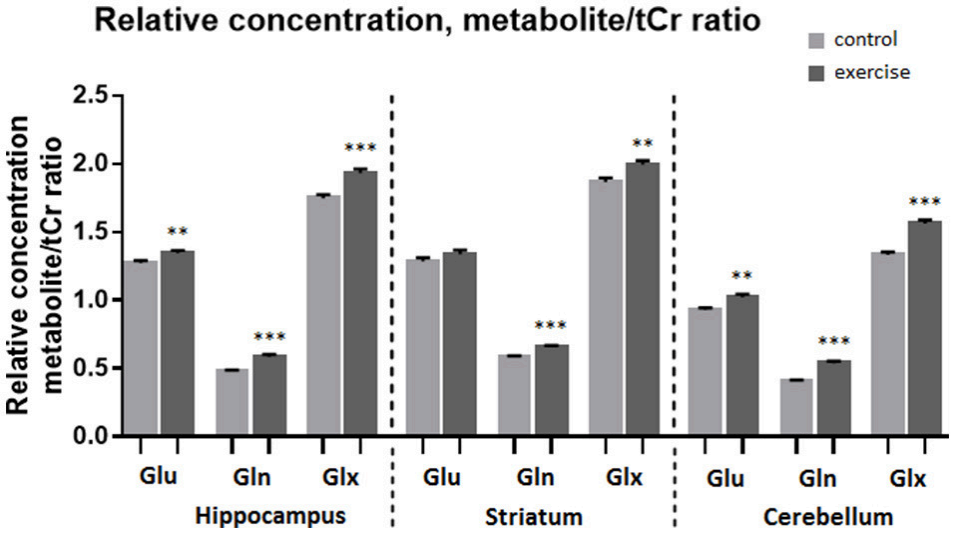
**Average relative concentration of Glu, Gln, and Glx in voxels centered on the hippocampus, striatum, and cerebellum before and after exercise**. Individual values of metabolite concentrations were scaled to total creatine concentrations. Mean ± SEM. ^**^P < 0.01, ^***^P < 0.001.

In the hippocampus Gln was increased by 21% when scaled to tCr (*p* < 0.001) and by 22% when scaled to water (*p* ≤ 0.001), Glu level was increased by 6% (to tCr, *p* < 0.005), and also by 6% (to water, *p* ≤ 0.05). Glx was increased by 10% when scaled to tCr (*p* < 0.001) and also by 10% when scaled to water (*p* ≤ 0.0005, Table 1, Figures 4, 5).

**Table 1:** **Average relative concentrations of Glu, Gln, and Glx (metabolite to water ratios and to tCr ratios) ± ***SD*** in the cerebellum, striatum, and hippocampus**.

		**Relative concentration, metabolite/water ratio**			**Relative concentration, metabolite/tCr ratio**		
		**Before exercise**	**After exercise**	***p***	**Before exercise**	**After exercise**	***p***
Glu	Cerebellum	9.00 ± 1.10	9.99 ± 1.08	0.0342	0.93 ± 0.06	1.02 ± 0.08	0.0049
	Striatum	8.90 ± 1.08	9.42 ± 0.58	ns	1.28 ± 0.10	1.34 ± 0.11	–
	Hippocampus	9.29 ± 0.86	9.83 ± 1.16	0.0342	1.27 ± 0.07	1.34 ± 0.06	0.0049
Gln	Cerebellum	3.91 ± 0.33	5.25 ± 0.50	0.0005	0.41 ± 0.04	0.54 ± 0.05	0.0005
	Striatum	4.03 ± 0.45	4.64 ± 0.36	0.0005	0.58 ± 0.04	0.66 ± 0.04	0.0010
	Hippocampus	3.47 ± 0.33	4.23 ± 0.47	0.0010	0.48 ± 0.05	0.58 ± 0.06	0.0010
Glx	Cerebellum	12.91 ± 1.30	15.23 ± 1.37	0.0010	1.33 ± 0.08	1.56 ± 0.10	0.0005
	Striatum	12.93 ± 1.45	14.06 ± 0.59	0.0093	1.87 ± 0.11	2.00 ± 0.10	0.0015
	Hippocampus	12.75 ± 1.08	14.06 ± 1.51	0.0005	1.75 ± 0.09	1.93 ± 0.10	0.0010

**Figure 6:**
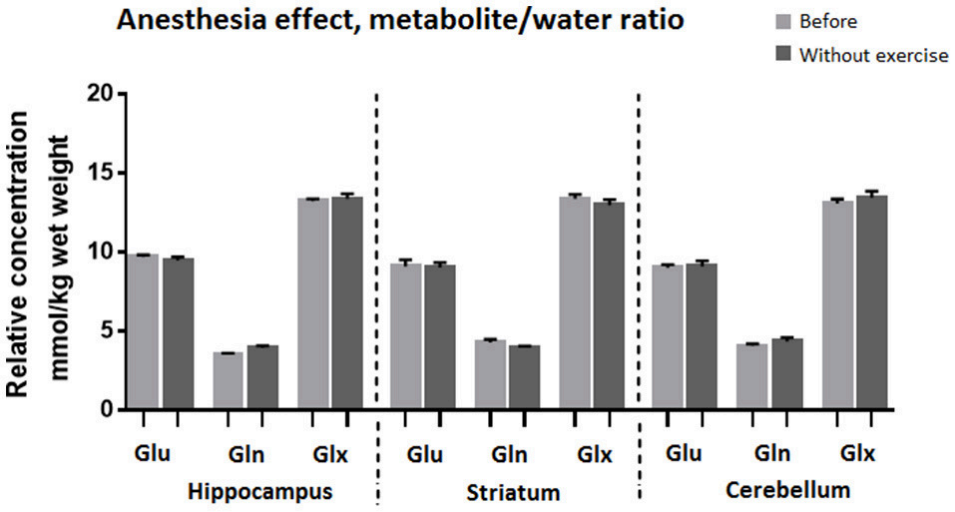
**Average relative concentration of Glu, Gln, and Glx in voxels centered on the hippocampus, striatum, and cerebellum after two sessions of anesthesia**. Individual values of metabolite concentrations were scaled to water.

**Figure 7:**
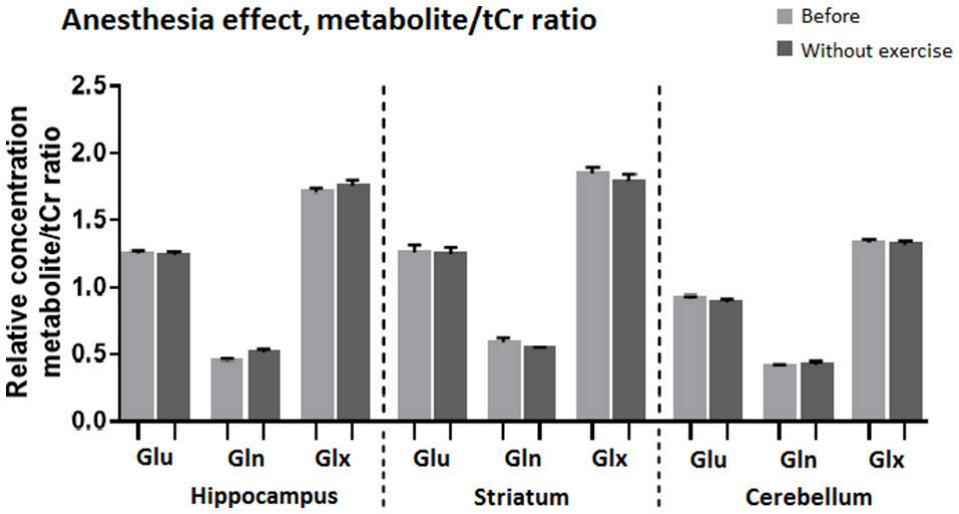
**Average relative concentration of Glu, Gln, and Glx in voxels centered on the hippocampus, striatum, and cerebellum after two sessions of anesthesia**. Individual values of metabolite concentrations were scaled to total creatine concentrations.

Comparison of MR spectra of the control group, recorded after the first and the second anesthesia run, revealed no significant changes (Figures 6, 7).

## Discussion

**Figure 8:**
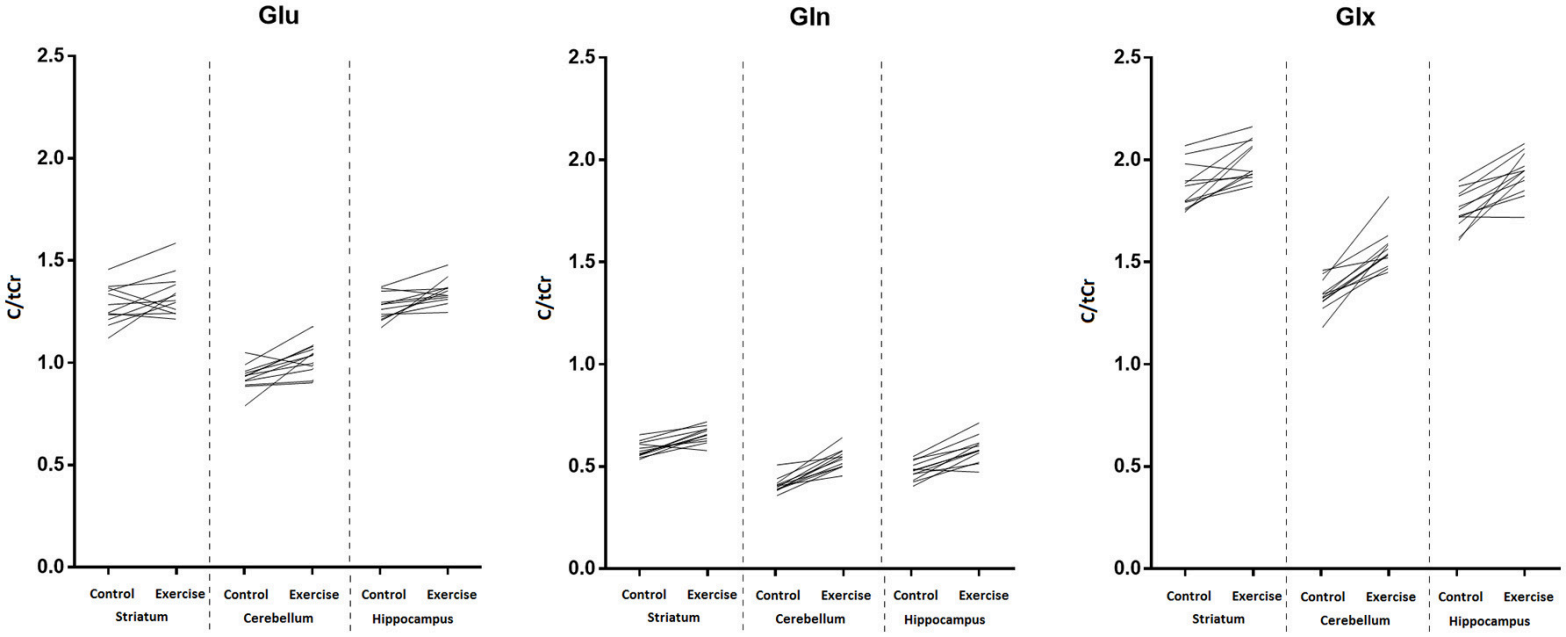
**Individual trends for Glu (left panel)**, Gln **(middle panel)**, and Glx **(right panel)** in the striatum, cerebellum, and hippocampus before and after exercise. Values were scaled to total creatine concentration.

The major finding of this study was that shortly after involuntary endurance exercise to exhaustion, increased glutamine (Gln), and combined glutamate-glutamine (Glx) signals were detected in the three VOIs located in regions of the rat brain supposedly activated during exercise, namely the hippocampus, striatum, and cerebellum. However, statistically significant increases in glutamate (Glu) signal were found only in the cerebellum and hippocampus. Increases in Glu appeared to be less consistent than those of Gln. In fact, although exercise-induced increase in Glu signal was statistically significant in two of the three VOIs (as evaluated by a non-parametric test), in some animals Glu signal actually decreased following exercise (Figure 8). Thus, our present data indicates that the two metabolic effects of exercise in the brain, increase in glutamine and increase in glutamate, result from independently regulated processes.

Before entering discussion on the possible physiological meaning of the results of the present study, let us analyze possible sources of error. From the point of view of the data integrity there are positive and negative consequences of conducting experiments on rats as compared to experimentation on humans. On one side rats of the Wistar strain are almost ideally standardized experimental mammals (Clause, 1993), therefore interanimal variability in response to exercise is expected to be low. On the other side, the rats had to be anesthetized before any MR imaging and spectroscopy run, which certainly will affect the resonance spectra. Highly significant differences in most ^1^H-MRS signals recorded at magnetic field of 9 T from the murine brain not exposed and exposed to 1.75% isoflurane were reported, including tCr higher by 3.8%, Glu higher by 7.6%, and higher Gln by 5.3% calculated from Table 1 in (Boretius et al., 2012). However, in the control experiments, in which two rounds of MR imaging under isoflurane anesthesia were separated by a period of rest of the duration similar to the period of exercise, we found no significant differences in ^1^H-MRS signals recorded during the first and the second imaging run. This allows us to conclude that neither anesthesia, nor the post-anesthesia stress likely present both in the control and in exercise experiments, is responsible for the changes detected after the exercise bout. The other possible source of error could be exercise-induced changes in the concentration of water and total creatine in the VOI. We were unable to find data concerning the behavior of brain water during exercise, but Dworak et al. (2007), who looked in the rat brain for the exercise-induced changes of adenosine and its metabolites with the use of chemcial assay methods, reported that they did found changes in neither creatine nor phosphocreatine.

The changes in the rat brain ^1^H-MRS spectra observed in the present study, which apparently are exercise-induced, are different from the results reported by Dennis et al. (2015) who noted significant lowering of Glu and Gln signals normalized to tCr following exercise in humans. These authors explained their finding by assuming that during exercise, when blood plasma lactate increases, the brain switches to use lactate instead of glucose as the major fuel for energy metabolism, therefore brain glucose uptake as well, as anaplerotic enrichment of the TCA cycle decreases. To support their hypothesis the authors quote reports showing that, indeed, during exercise the brain may use lactate as a fuel alternative to glucose. Although our present data did not show significant lactate resonance signals following exercise, we cannot exclude that increased availability of lactate in arterial blood makes it possible to use it as a preferred fuel for brain energy metabolism. Also, in our opinion, switching to lactate as a major fuel for brain energy metabolism shall not suppress anaplerosis, because lactate will be converted to pyruvate and enter the TCA cycle as acetyl-coenzyme A. Our present data are also different from the metabolic response to chronic voluntary exercise in mice reported by Biedermann et al. (2012). This is likely related to the difference between acute exercise, which leads to a transient but ^1^H-MRS detectable disequilibrium and chronic exercise that creates a new metabolic equilibrium in brain tissues. Further studies of the metabolic effects of exercise on the human brain with the use of ultra-high magnetic fields are indicated to confirm translatability of rodent ^1^H-MRS data to humans.

On the other hand, our results are comparable with those reported previously by Maddock et al. (2011). These authors noted transient increases in Glx signal following exercise, and interpreted them as a result of the activity-dependent increase in brain Glu content. Such interpretation may be correct because in humans brain activation during vigorous exercise has been reported to provoke a “surplus” uptake of glucose relative to the oxygen uptake (Dalsgaard, 2006). This could be the source of anaplerotic enrichment of the tricarboxylic acid cycle (TCA) and the consequential increase in total brain glutamate.

According to the current understanding (Schousboe et al., 2013, 2014; Verkhratsky et al., 2015) glutamine in the brain is generated by glutamate amination catalyzed by glutamine synthetase (GS), an enzyme that is expressed selectively in astrocytes. Glutamate, the substrate in this reaction comes from clefts in the excitatory synapses, from where it is taken up by excitatory amino-acid transporters (EAATs) which serve to terminate the excitatory signal. The main function of the brain's glutamine pool is to serve as the functional reserve for the generation of glutamate. The glutamine synthesized in astrocytes leaves via the system N transporter (SN1) and diffuses through extracellular space to glutamatergic neurons, where it is then taken up by the system A transporters (SAT1 and SAT2) and subsequently converted back to glutamate by phosphate-activated glutaminase (PAG). This last reaction completes the glutamate-glutamine cycle, which is thought to recirculate a substantial part of the glutamate neurotransmitter pool. However, as already mentioned, MRS recording provides the signal generated by the nuclei present in all compartments in the VOI, which means that this technique neither distinguishes between resonance signals originating from neurons and glia, nor between resonance signals of neurotransmitter and non-neurotransmitter pools of Glu. The result is always a weighted mean concentration of a given metabolite across all compartments in the VOI. Consequently, a mere flux of a given metabolite from one compartment to the other, e.g., from neurons to glia, will not cause any change in the MRS signal. A change in the MRS signal is a result of a change in metabolite concentration (within the VOI), and it could be caused by net inflow and/or synthesis, or net outflow and/or degradation of the metabolite^1^.

Although both glutamine and glutamate are abundant in the brain (where they reach milimolar concentrations), the level of glutamate is two to three times higher than the level of glutamine (Erecińska and Silver, 1990). It is commonly believed that in the brain, the majority of glutamate is present in neurons and the majority of glutamine is present in astroglia. Extracellular space within the brain consists of synaptic and extrasynaptic compartments. *In situ* brain microdialysis usually provides an estimate of glutamate in the extrasynaptic compartment (Drew et al., 2004), whereas at the same time glutamate concentration inside synaptic clefts, which are very narrow, may momentarily reach several milimoles (Bergles et al., 1999), and glutamate is very quickly cleared by EAATs. We may assume that the flux of glutamate from neurons to astrocytes occurs mostly through very narrow synaptic clefts, and that EAATs-mediated glutamate uptake to the astrocytes is much more efficient than glutamine transport from astrocytes to neurons through the much wider extrasynaptic extracellular spaces.

Acute high intensity exercise increases excitatory synaptic activity in brain areas involved in trafficking exercise-relevant signals. The expected result is the release of large amounts of glutamate from vesicles in presynaptic terminals to the synaptic clefts, from where they are quickly removed by the very efficient EAATs and enter astrocytes. An increase in the Gln signal occurs because the gradient of glutamine concentration across extrasynaptic ECF, i.e., between SN1 and SAT1/2 transporters, is flat compared to the steep glutamate gradient between presynaptic membrane and postsynaptic membranes equipped with very highly active EAAT transporters. Therefore, glutamine entry to neurons is slower than glutamate entry to the astrocytes. The other process which may contribute to the increase in Gln concentration is the increased need for detoxification of brain ammonia which occurs through conversion of Glu to Gln. High levels of ammonia in the brain during exercise is a result of the increased production of ammonia by contracting muscles and increased cerebral uptake (Wilkinson et al., 2010).

**Figure 9:**
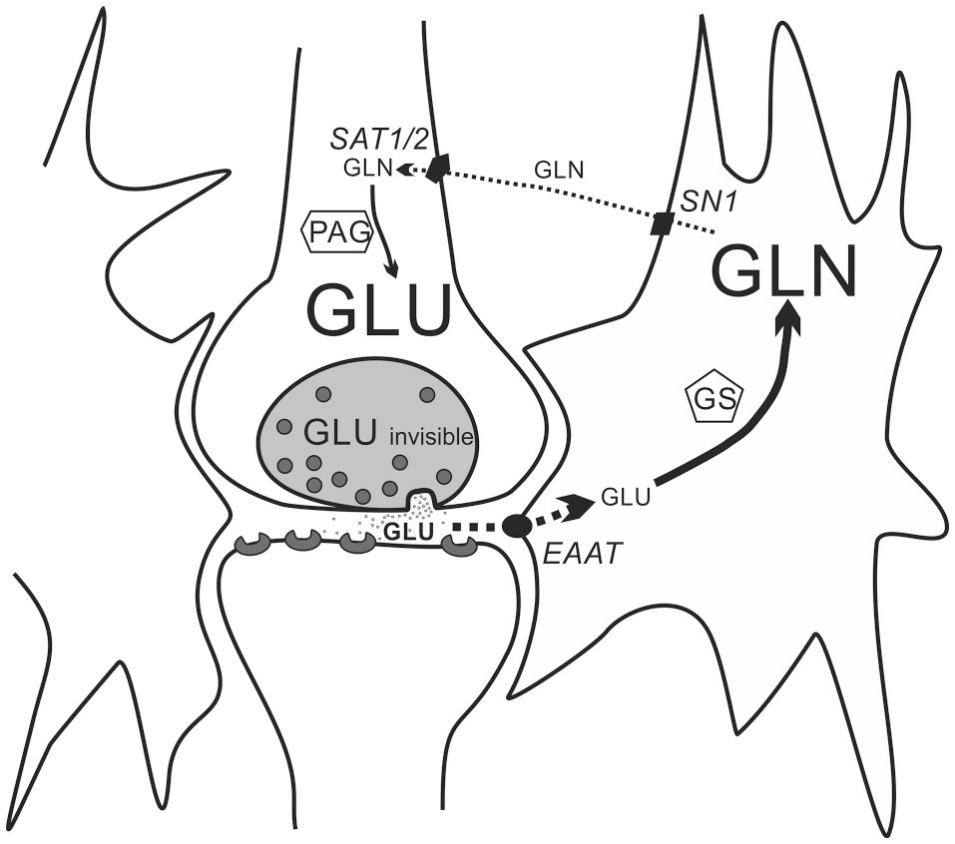
**A simplified scheme of the glutamate-glutamine cycle**. Within a glutamatergic synapse, molecules of the excitatory neurotransmitter glutamate are released from presynaptic vesicles to the synaptic cleft, and shortly after their release they are taken up by the very active EAATs excitatory amino-acid transporters located in membranes of astrocytes. Glutamate taken up by the astrocytes is quickly aminated by glutamine synthetase (GS) to glutamine. Glutamine exits astrocytes through the SN1 transporter proteins to the extracellular space and diffuses through the extracellular space to the surface of glutamatergic neurons, where it is taken up by SAT1 and two transporters. In the glutamatergic neurons glutamine is converted by phosphate-activated glutaminase (PAG) back to glutamate. Because of the difference in diffusion distance (a very narrow synaptic cleft compared to an average distance between an astrocyte membrane and a membrane of a glutamatergic neuron) and in activity of membrane transporter proteins (the activity of EAATs is much higher that of the SN1 and SAT1/2) astrocytic uptake of glutamate and its conversion to glutamine (thick sold line with arrow) is faster than reciprocal transport of glutamine from astrocytes to neurons and its conversion back to glutamate (thin broken line with arrows). For simplicity connections between the glutamine-glutamate cycle and other cellular metabolic pathways are neglected.

The summary of our hypothesis is presented in Figure 9. As previously mentioned, the increased turnover of the glutamate-glutamine cycle by itself would not change the Glx signal because the increase in Gln should be balanced with the decrease in Glu. The increase in Glu observed in some brain areas during exercise likely results from activation of anaplerosis which enriches the brain's glutamate pool through the TCA cycle and overshoots the Glu decrease due to its conversion to Gln. Although it has been stated that in the brain anaplerosis shall be matched by its opposite, cataplerosis (Olsen and Sonnewald, 2015), this shall hold true for equilibrium situations. In our experiments the relatively short exercise run, although certainly longer and more exhaustive than those in human ^1^H-MRS experiments, could have created a disequilibrium visible as a synchronous increase in Glx, Gln and Glu.

Finally, we would like to comment on a possible relevance of our findings to the issue of the so-called central motor fatigue. Although factors determining limitation of endurance exercise have been classically sought in the cardio-pulmonary and muscle metabolic capacity, already at the end of nineteenth century the concept of central (mental) fatigue that could be distinguished from peripheral fatigue has been proposed by the Italian physiologist Alberto Mosso (see Di Giulio et al., 2006). More than 100 years later, in his review devoted to the spinal and supraspinal factors in human muscle fatigue, Gandevia (2001) discussed a possible role in the central fatigue phenomenon of changes in some CNS neurotransmitter systems, namely serotonergic, dopaminergic and GABA-ergic. Two years later Kayser (2003) remarked that any voluntary exercise starts and ends in the brain, and suggested that CNS, which integrates inputs from various sources, is ultimately limiting exercise performance in order to prevent jeopardizing the integrity of the organism. We think that the same shall hold true for a forced exercise. However, as far as we know, glutamatergic neurotransmission has never been mentioned in the context of the central fatigue phenomenon.

Recently, Hou et al. (2016) elicited a motor fatigue state in human healthy volunteers by exhaustive physical exercise and used functional magnetic resonance imaging (fMRI) to visualize neural activation response to a standard paradigm of hand flexion and extension movements. Under the effect of motor fatigue the neural responses to these simple movements in the sensorimotor cortical areas as well, as in the thalamus and basal ganglia were highly significantly attenuated compared with those recorded before the exhaustive physical exercise. In the other recent paper Sonnay et al. (2016) reported that, compared to resting state, the rat cortex stimulated with electrical stimulation of the paws, a paradigm typically used for evoking fMRI response, exhibited increased glutamate-glutamine cycle along with enhancement of neuronal and glial oxidative metabolism. Considering the above we suggest that the disequilibrium in the glutamate-glutamine cycle, signs of which we have observed in our experiments following acute endurance to exhaustion, may be an important contributor to the central motor fatigue phenomenon.

## Ethics statement

IV local ethics committee in Warsaw, Poland.

## Author contributions

MS, MF, JO, MW, PB, JL, and PG contributed to the study design. PG, MS, and MW contributed to acquirement of ethical approval. MS, MF, JO, and MW contributed to data collection. MS, MF, JO, MW, JL, PB, and PG analyzed the data, interpreted the data, and drafted the initial manuscript. All authors revised and approved the final version of the manuscript. MS, MF, and PG are guarantors of the manuscript and take full responsibility for the work as a whole, including the study design, access to data, and the decision to submit and publish the manuscript.

### Conflict of interest statement

The authors declare that the research was conducted in the absence of any commercial or financial relationships that could be construed as a potential conflict of interest.

## Footnotes

^1^In certain conditions of MR measurement the estimation of metabolite concentration from the spectrum may turn to be significantly lower than the result of the chemical assay of this metabolite in a homogenized tissue sample. This occurs when, due to restrictions of molecular diffusion (for example due to membrane binding), a fraction of chemically detectable metabolite turns out to be “MRS-invisible.” The partial resonance “invisibility” may be the case for glutamate in the brain, because a substantial amount of this amino-acid is tightly packed to presynaptic vesicles. Indeed, when MR measurement has been performed with the long echo times (120 ms), the “invisible” glutamate fraction amounted to 1/3 of total chemically detectable brain glutamate (Kauppinen et al., 1994). However, in our experiments, the influence of restriction of glutamate diffusion was negligible because of much shorter echo time used in MR signal acquisition (20 ms).

## Abbreviations

^1^H-NMR: proton magnetic resonance
Ala: alanine
Asp: aspartate
CNS: central nervous system
Cr: creatine
CSF: cerebrospinal fluid
EAATs: excitatory amino-acid transporters
ECF: extracellular fluid
fMRI: functional magnetic resonance imaging
GABA: γ-aminobutyric acid
Glc: glucose
Gln: glutamine
Glu: glutamate
Glx: combined glutamine-glutamate signal
GPC: glycerophosphocholine
PCh: phosphocholine
GS: glutamine synthetase
GSH: glutathione
NIH: National Institutes of Health
Ins: *myo-*inositol
Lac: lactate
MR: magnetic resonance
MRS: magnetic resonance spectroscopy
NAA: N-acetylaspartate
NAAG: N-acetyl aspartate glutamate
NMR: nuclear magnetic resonance
PAG: phosphate-activated glutaminase
PCr: phosphocreatine
SAT1, SAT2: system A transporters
Scyllo: scyllo-Inositol
SN1: system N transporter
Tau: taurine
tCho: signal of choline compounds
tCr: creatine and phosphocreatine
VOI: volume of interest.
